# Misrepresentation

**Published:** 1882-03

**Authors:** Ad. Lippe

**Affiliations:** Philadelphia


### MISREPRESENTATION.

AD. LIPPE, M. D., PHILA.

"We know of no practice more contemptible than that of deliberate misrepresention, and unfortunately there are those who seem to have little ability beyond. It is such as these that have done and are doing our school so much harm, and its survival, regardless of these "*friends*," is an indication of strength which will withstand anything! Fortunately, these men are so largely in the minority that their influence is but trifling, and the bragging assumption with which they arrogate to themselves all the knowledge there is of Homœopathy, disgusts the majority and they discard them altogether!"

The above are the introductory sentences of a paper on "*Misrepresentation*," published in the *N. Y. Medical Times* (an avowedly *eclectic* journal), Jan. 1, 1882. When the learned editor speaks of "Our School," he can but have reference to the school which his journal fully and ably represents—the eclectic. This journal was formerly known as the *Homœopathic Times*, but as it gradually drifted away into eclecticism the editors, with a praiseworthy honesty, dropped what they considered a "nickname," and became the outspoken antagonists of Hahnemann's healing art. It becomes our unpleasant duty to prove our proposition, viz.: that the *Medical*

*Times* represents the eclectic school, notwithstanding their absurd claim of representing the homœopathic school, and their lecturing and abusing the followers of Hahnemann, as is evident by the sentences following the one above quoted. Honest is the *Medical Times*, and for their honesty we bestow on the journal and its learned editors all the praise honesty deserves. Honestly and without reserve the journal utters its "Declaration of Principles" on page 313. They give us their "three foundation axioms," viz.:

1. The careful taking of the case both for diagnosis pathologically and for the purpose of the choice of treatment.
2. The strict individualization of the case as to means to be employed.
3. The selection of the means, in accordance with the exigencies, whether remedial or otherwise, conforming these agents to the most approved modes of experience, avoiding undue importance to any, and always holding uppermost the greatest good to our patient."

The three foundation axioms of Homœopathy have been, are, and will forever be:

1. The Law of the Similars.
2. The single remedy.
3. The minimum dose of a proved remedy.

The homœopathist is taught that his highest and only calling is to cure the sick by strictly applying these three fundamental principles to each individual case.

By what compass is this new medical school, which the *Medical Times* represents, guided? There is surely no falsification in the statement here made; that the true foundation axiom of the new school can not be foisted on Homœopathy.

If they stand for axioms, the learned editors might have been kind enough to add some explanatory notes to them, for the benefit of men who have accustomed themselves to follow the inductive method of the founder of the homœopathic healing art. After such explanatory notes making it clear to the dullest intellect what these philosophers really are driving at, criticism might be in place, but at present it is hard to tell the true inwardness of their axioms; as far as a common intellect can comprehend axiom 1, we must say that Homœopathy *never* taught that the sick should be examined for a diagnosis pathologically; as if we ever could apply the law of the Similars to a pathological condition—that is allopathy to a fault. Where, pray, did Hahnemann teach us to treat pathological conditions or sick physiology? And what, pray, does the learned editor imply when he suggests that after duly diagnosticating the case pathologically, we should then and on that account, after our finding, choose the treatment? We then have a choice of treatment? Have the homœopathists? NO? Have the eclectics? *Yes!* The homœopathist, if he is an honest man, if he adheres to the fundamental principles of the school he belongs to, after collecting all the objective and subjective symptoms of the sick, seeks the similar remedy among the proved drugs. The eclectic, as the *Medical Times* has it, diagnosticates disease, and then he resorts to a remedy, which to his knowledge has at times and under certain, to him unknown, circumstances cured that form of disease; or he palliates, not hesitating to use his favored pain-killer, the hypodermic injection of *Morphia;* or he diagnosticates Malaria and administers massive doses of *Quinine;* or he flies to isopathic remedies—just as his fancy dictates, all for the greatest good of the sick!

How often, with what unsurpassed patience, with what great politeness have we not humbly prayed for "clinical reports" by the philosophers and healers who brag and boast of superior results outside of strict Homœopathy. We pray again:—Let us hear from you, who are wiser than was our Master; let us hear from you before you, imagining yourself in a "large majority," refuse a place in the profession to men who, you say, have little practical experience; let us compare "*Results!*" We again join hands with you when you say, "Science knows no other bondage than a submission of its votaries to *truth.*" You have very often doubted the correctness of Hahnemann's teachings, doubted the general applicability of the Law of Similars and its corollaries. The burden of the proof lies with you; others, not a small number, have testified in the affirmative thousands of times, that the best success possible in healing the sick is obtained by practicing Homœopathy as taught by its founders. Now, if you seek the *truth* and care to be considered *honest*, please let us know one single case treated unsuccessfully under the strict homœopathic law and rules, and afterward cured by eclectics; you will thereby establish the gauge by which your ability to sit in judgment over Homœopathy may be shown.

